# Perceptions of air pollution from stubble burning and its health risks in Punjab, India

**DOI:** 10.1038/s41598-025-21235-8

**Published:** 2025-10-27

**Authors:** Zhesi Yang, Kayo Ueda, Tomohiro Umemura, Kazunari Onishi, Hiroaki Terasaki, Tomoki Nakayama, Yutaka Matsumi, Kamal Vatta, Hikaru Araki, Sachiko Hayashida, Prabir K. Patra

**Affiliations:** 1 Research Institute for Humanity and Nature, Kyoto, 6038047 Japan; 2 Department of Global Health Policy, Graduate School of Medicine, The University of Tokyo, Tokyo, 1130033 Japan; 3 Department of Hygiene, Graduate School of Medicine, Hokkaido University, Kita 15jo, Nishi 7chome, Sapporo, Hokkaido 060-8638 Japan; 4 Aichi Medical University School of Medicine, Nagakute, 480115 Japan; 5 Division of Environmental health, Graduate School of Public Health, St Lukes International University, Tokyo, 1040045 Japan; 6 Faculty of Engineering, University of Fukui, Fukui, 9108507 Japan; 7 Faculty of Environmental Science, Nagasaki University, Nagasaki, 8528521 Japan; 8 Institute for Space-Earth Environmental Research, Nagoya University, Nagoya, 4648601 Japan; 9 Department of Economics and Sociology, Punjab Agricultural University, Ludhiana, Punjab 141004 India; 10 Research Institute for Global Change, JAMSTEC, Yokohama, 2360001 Japan; 11 Nara Women’s University, Nara, 6308263 Japan

**Keywords:** Risk perception, Air pollution, Stubble burning, Health risk, Public health, Environmental social sciences, Sustainability, Environmental impact

## Abstract

In the state of Punjab, India, where a rice-wheat double cropping system is prevalent, smoke from paddy stubble burning deteriorates air quality in the area and downwind regions. To assess citizens’ perception about air pollution, its health risks, and attitude toward stubble burning, a semi-structured interview was conducted with 2202 households in 22 districts of Punjab between August 2020 and January 2021. We explored household- and district-specific factors affecting each dimension of health risk perception using the random-intercept logistic regression model. Among all households, 46% of respondents perceived air pollution in Delhi as “severe” while only 25% perceived that in Punjab areas as “severe”. Although 60% of respondents noted that smoke from stubble burning did not affect their health or that of their families, more than 65% considered stubble burning to be a major issue. Respondents whose family members have health issues or who are aware of the health risks of air pollution were more likely to perceive smoke from stubble burning is harmful to health. Our findings suggest that while households are aware of the poor air quality in Delhi, they did not recognize stubble burning to be the main cause – in part due to lack of observational evidence.

**Supplementary Information:**

The online version contains supplementary material available at 10.1038/s41598-025-21235-8.

## Introduction

The Global Burden of Disease study reported that over 1.6 million premature deaths were attributed to long-term exposure to air pollution in India^1^. Globally, the Indo-Gangetic Plain (IGP) is one of the regions most severely affected by high levels of fine particulate matter (PM_2.5_)^2^, and the Delhi national capital region (NCR), which is within the IGP, has often been ranked amongst the most polluted cities in India and even worldwide^3^. This is particularly evident in October and November when severe pollution episodes typically occur^4,5^, and persists through February of the following year^6^. The PM_2.5_ with aerodynamic diameter of 2.5 μm can enter the human respiratory and cardiovascular systems.

Studies have suggested that large quantity of smoke emitted from open crop residue burning in the north-western states of India is responsible for some of the extreme air pollution episodes during the post-monsoon period^7–10^. Punjab, located upwind of the IGP in the October-November months, has adopted a rice-wheat double cropping system in the 1970s^11,12^. While the double-cropping system has increased grain production and contributed a significant proportion to the national food grain pool (24.2% and 41.5%, respectively, in 2015)^11^, it has also imposed a tremendous environmental burden. Burning crop residue emits substantial amounts of smoke, which deteriorates air quality not only locally but also in downwind regions. Furthermore, intense cultivation in the region leads to soil degradation through nutrient loss and contributes to depletion of groundwater^12^.

The government of India has implemented several policies to reduce crop residue burning, such as monitoring field burning via satellite, encouraging the ex-situ use of crop residue in industries, promoting mechanized sowing machinery for stubble management, and providing financial support^13,14^. However, the government policies have not yet achieved any improvement in air quality of Delhi-NCR^15^ or a significant reduction in crop residue burning^16^, primarily because farmers are reluctant to adopt more sustainable methods of managing crop residue due to economic constraints and time limitations^17^.

In this context, it would be beneficial to understand how farmers in this region, who are involved in crop residue burning, perceive air quality and the health risks posed by air pollution to themselves and their families. Many studies have evaluated how people perceive the risk of air pollution in relation to individual behavioral changes^18^. The perception of air pollution has an impact on people’s behaviors, socio-technical vulnerabilities, and governance, while social behavior and policy interventions can influence the degree and spatial extent of air pollution^19^. However, few studies have specifically focused on public perception of air pollution due to crop residue burning^20^. The objective of this study is to explore perceptions of and knowledge about air pollution, health risks involved, and attitudes toward stubble burning.

## Results

### Sociodemographic characteristics of respondents

The characteristics of respondents are summarized in Table 1. Among 2202 households, most respondents were adult males (98.9%) and non-smokers (96.5%). More than half (51.6%) of the respondents were aged between 40 and 59 years, and the majority (63.3%) have completed secondary school. The household annual income ranged from 5000 to 6,250,000 Indian National Rupees (INR) with a mean of 479,451 INR. In addition, 49.8% households had more than five family members. The prevalence of households reporting health problems within household was lower in the stubble burning season than that in other seasons (15.3% vs. 16.8%, McNemar’s Chi-square statistic = 7.78; *p* < 0.05). Over 40% of respondents did not know that air pollution could increase the risk of respiratory and cardiovascular diseases.

**Table 1:** Individual/household characteristics of respondents (*n* = 2202).

Characteristics	Participants, *n* (%)
*Sex*	
Male	2176 (98.8)
Female	26 (1.2)
*Age (years)*	
20–39	434 (19.7)
40–59	1136 (51.6)
60-	632 (28.7)
*Smoking*	
Yes	78 (3.5)
No	2124 (96.5)
*Number of household members*	
1–2	102 (4.6)
3–4	1003 (45.5)
5–6	790 (35.9)
6-	307 (13.9)
*Education level*	
Primary or lower	656 (29.8)
Secondary	1394 (63.3)
Diploma and above	152 (6.9)
*Annual income (INR)*	
< 144,941	525 (23.8)
144,941 − 300,000	619 (28.1)
300,001-600,000	452 (20.5)
> 600,000	503 (22.8)
NA	103 (4.7)
*Health condition*	
Well	1728 (78.5)
Sick	420 (19.1)
NA	54 (2.4)
*“Having any family members who have health problems in stubble burning season in 2019?”*	
Yes	337 (15.3)
No	1830 (83.1)
NA	35 (1.6)
*“Having any family members who have health problems in other season in 2019?”*	
Yes	371 (16.8)
No	1792 (81.4)
NA	39 (1.8)
*Knowledge about health risks of air pollution* *“Do you know air pollution increase the risk of respiratory and cardiovascular diseases?”*	
No problem	969 (44.0)
Yes, a little	703 (31.9)
Yes, very much	499 (22.7)
NA	31 (1.4)

### Perceptions of air pollution in Punjab and Delhi

Approximately 46% of respondents perceived air pollution in Delhi as “severe,” while only 24.5% perceived air pollution in Punjab areas as “severe” (Table 2). The proportion of respondents perceiving air pollution as “severe” varied across districts (Fig. S1). In response to questions about the causes of air pollution in Delhi, over 75% of respondents believed that it was caused by air pollutants emitted from Delhi NCR itself. Fewer than 30% thought that stubble burning was a significant contributor to air pollution in Delhi. In total, 19.4% of households reported illness throughout the year. Regarding health risk perception, 58.2% of respondents stated that smoke from stubble burning did not affect their health or that of their families. When asked about their attitude toward stubble burning in Punjab, more than 65% of households considered it a “Big problem, and have to stop now,” followed by “Some problem, but cannot stop” (17.1%), “Not interested” (10.6%), and “Not a problem” (4.2%).

**Table 2:** Questionnaire asking the perception about air quality and its health risks.

Variable	Number (%)
*Perceived air pollution in Delhi* *“How do you think about air pollution in Delhi in November 2019?”*	
No problem	220 (10.0)
Little	257 (11.7)
Medium	676 (30.7)
Severe	1022 (46.4)
NA	27 (1.2)
*“What do you think about the cause of air pollution in Delhi? (multiple choices are allowed)”*	
Pollutants from Delhi NCR	1655 (75.2)
Stubble burning in Haryana	587 (26.7)
Stubble burning in Punjab	113 (5.1)
Others	290 (13.2)
NA	26 (1.2)
*Perceived air pollution in Punjab* *How do you think about air pollution in Punjab in November 2019?*	
No problem	229 (10.4)
Little	738 (33.5)
Medium	672 (30.5)
Severe	540 (24.5)
NA	23 (1.0)
*Perceived health risk of smoke from stubble burning* *“Do you think smoke from stubble burning affects you and your family’s health?”*	
No problem	1281 (58.2)
Yes, a little	468 (21.3)
Yes, very much	433 (19.7)
NA	20 (0.9)
*Attitude toward stubble burning* *“What do you think about stubble burning in Punjab?”*	
No problem, and do not have to stop	93 (4.2)
Some problems, but cannot stop	377 (17.1)
Big problem, and have to stop now	1434 (65.1)
No interest	234 (10.6)
NA	64 (2.9)

### Individual/household factors and perceptions

Results of the logistic regression model indicate that age was associated with perceptions about air pollution (Table 3; see also Methods). Respondents aged 40 and older had a higher odds of perceiving the air pollution in Delhi and Punjab as severe compared with those under 40. Educational level, household size, and income level were not significantly associated with any perception variables. Households with members who had health issues were substantially more likely to perceive smoke from stubble burning as harmful to their health (OR 45.27, 95% CI 16.34, 125.43) and were also more likely to consider stubble burning as a big problem in Punjab (OR 2.04, 95% CI 1.11, 3.74). Respondents with knowledge about the health risks of air pollution were more likely to perceive air pollution in Delhi as severe (OR 1.99, 95% CI 1.45, 2.74), believe that smoke from stubble burning affected their health (OR 24.82, 95% CI 15.58 39.51), and regard stubble burning as a big problem in Punjab (OR 3.19, 95% CI 2.29, 4.45). In the sensitivity analysis using district as dummy variable, the results were similar to those from the main model (Table S1).

**Table 3:** Odds ratios (ORs) of individual factors on perception of air quality and its health risks including district level variables.

Explanatory variable	Perceived air pollution in Delhi	Perceived air pollution in Punjab	Perceived health risk of smoke from stubble burning	Attitude toward stubble burning
Individual-/household-level variables	“Severe” vs. “No problem”/“Little”/“Medium”		“Yes, very much”/“Yes a little” vs. “No problem”	“Big problem, and have to stop now” vs. other responses
*Age*				
Less than 40 year (Reference)	1	1	1	1
40–59 year	1.50 (1.04, 2.15)*	1.77 (1.11, 2.83)*	1.46 (0.90, 2.37)	0.72 (0.51, 1.02)
60 year and over	1.43 (0.94, 2.18)	1.76 (1.05, 2.97)*	0.87 (0.49, 1.54)	0.79 (0.52, 1.18)
*Educational status*				
Primary (reference)	1	1	1	1
Secondary/diploma	1.22 (0.91, 1.64)	0.76 (0.54, 1.08)	0.91 (0.60, 1.38)	1.21 (0.90, 1.62)
Graduate/above	1.27 (0.65, 2.50)	1.74 (0.78, 3.90)	0.85 (0.36, 2.05)	1.22 (0.66, 2.26)
*Household members*				
1 or 2 (reference)	1	1	1	1
3 or 4	0.74 (0.39, 1.41)	1.51 (0.69, 3.3)	0.90 (0.37, 2.17)	0.75 (0.39, 1.45)
5 or 6	0.98 (0.51, 1.87)	1.30 (0.59, 2.86)	0.61 (0.25, 1.52)	0.59 (0.30, 1.13)
7 and over	0.79 (0.39, 1.60)	0.80 (0.34, 1.90)	0.63 (0.23, 1.67)	0.50 (0.24, 1.00)
*Household income*				
-144,940 (Reference)	1	1	1	1
144,941–300,000	0.78 (0.54, 1.12)	0.80 (0.53, 1.23)	1.33 (0.80, 2.20)	0.86 (0.60, 1.23)
300,001–600,000	0.69 (0.46, 1.03)	1.09 (0.68, 1.74)	1.17 (0.67, 2.03)	0.88 (0.59, 1.30)
600,001-	0.89 (0.58, 1.35)	0.90 (0.54, 1.51)	0.86 (0.47, 1.57)	1.05 (0.70, 1.56)
*Health condition of household*				
Well (reference)	1	1	1	1
Sick	0.89 (0.49, 1.59)	1.39 (0.68, 2.84)	45.27 (16.34, 125.43)*	2.04 (1.11, 3.74)*
Knowledge				
No (reference)	1	1	1	1
Yes	1.99 (1.45, 2.74)*	1.27 (0.84, 1.93)	24.82 (15.58, 39.51)*	3.19 (2.29, 4.45)*
District-level variables^†^				
Burning area proportion	0.97 (0.91, 1.04)	1.02 (0.90, 1.16)	1.02 (0.95, 1.10)	1.03 (0.98, 1.07)
Fire detection count^‡^	1.52 (0.44, 5.23)	0.76 (0.08, 7.24)	0.67 (0.19, 2.32)	0.52 (0.22, 1.19)

*P < 0.05.

^†^OR per a unit increase is shown.

^‡^Fire detection counts were transformed using a logarithmic scale.

### District-level characteristics and perceptions

Proportion of burnt area in 2020 and monthly total fire detection counts during October and November in 2018 and 2019 for each district are shown in Fig. S2 (see Methods for data source). These are simple proxies for crop residue burning. We examined both burnt area and log-transformed fire detection counts in the model, but no significant association was found with any of the dependent variables (Table 3).

## Discussion

We assessed perceptions of households in Punjab regarding air pollution and stubble burning in a cross-sectional survey. A significant portion of respondents viewed air pollution in Delhi as severe, while fewer perceived it as severe in Punjab. Additionally, a smaller percentage attributed the cause of air pollution in Delhi to stubble burning. Middle-aged and older respondents were more likely to consider air pollution to be severe in Punjab. Household health conditions and knowledge about health risks also played key roles in shaping perceptions of air pollution and health risks associated with stubble burning. At the district level, neither fire detection counts nor the extent of burnt areas showed significant associations with respondents’ perceptions of air quality or health risks.

### Perceptions of air quality in Delhi and Punjab, and its health risks

Over 45% of respondents considered air pollution in Delhi to be severe, while 30.7% considered it to be moderate. On the other hand, fewer respondents believed air pollution in Punjab to be severe. Delhi frequently experiences severe air pollution, ranking among the worst globally in terms of air quality^21^. Media coverage of Delhi’s air quality has further heightened public awareness^22^. Several studies have demonstrated the link between air pollution in Delhi and crop residue burning in neighboring states during the stubble burning season^4,10^. However, the majority of respondents did not consider Delhi’s pollution coming from outside of Delhi-NCR, such as Punjab. This difference in perception could be attributed to cognitive dissonance^23^. Most respondents were farmers who might have been aware that their agricultural activities contribute to air pollution via burning smoke. This conflicting awareness may have led to a distorted perception of the problem. Besides, cognitive dissonance may have influenced the perceived health risks. For example, some respondents may have been reluctant to acknowledge the health risk associated with air pollution from stubble burning, which could have resulted in an underestimation of the proportion of respondents who believe health risk of air smoke from stubble burning. Moreover, regardless of its potential health impacts, respondents may consider stubble burning a problem for agriculture—such as soil degradation and water pollution—and believe that something needs to be done about it.

### Factors affecting perceptions of air pollution and its health risks

Previous studies have suggested that perceptions of air quality risk are influenced by sociodemographic factors such as gender, age, education, knowledge, and health status^24,25^. In the present study, respondents aged 40 and older were more likely to perceive the air quality in Delhi and Punjab as poor. This is consistent with a study conducted in Chandigarh, which found that older individuals perceived greater health impacts from air pollution, possibly due to age-related chronic illnesses^26^.

Contrary to previous reports, neither educational status nor household income was associated with perceived air pollution in the present study. For example, a study in California, USA, found that education level and age did not significantly influence perceptions of air pollution^27^. However, studies conducted elsewhere have found that higher education is often linked to better knowledge of the risks of air pollution^28^.

Approximately 65% of respondents considered stubble burning to be a significant problem that must be stopped. This perception was associated with health problems among household members and knowledge about health risks associated with air pollution. Existing literature supports the idea that understanding the environment positively influences attitudes and behaviors^29,30^. This highlights the need for improved environmental health literacy. Organizing seminars or classes in local communities or schools could help households become more aware of air pollution and the importance of taking protective measures^31^. Additionally, providing better access to air quality information through straightforward and easily comprehensible channels would help residents understand and visualize the impact of air pollution more effectively^26^. Research suggests that the success of efforts and policies aimed at addressing environmental issues depends on public awareness and support for environmental protection^32^.

We examined the association between district-level variables, such as fire detection counts and burnt areas, as proxies for the extent of stubble burning. The extent to which stubble burning contributes to air pollution or the amount of smoke released into the atmosphere depends on density of stubble on the field, fuel burned fraction and various environmental conditions, such as, soil and stubble wetness, humidity and wind speed. Moreover, once PM_2.5_ is emitted into the atmosphere, it can persist for several days and thus spread across districts and states through prevailing winds. However, none of these were associated with any of the dimensions of perception of air pollution from stubble burning. Many studies have shown that air pollution levels affect perceptions of air pollution^33–35^. Those studies reported on the degree of air pollution and air quality index, without measurements from regions of intense stubble burning prior to 2022^5^. Air pollution levels within a district may be influenced not only by local emission sources but also by sources outside the district and prevailing wind directions, s and thus may not necessarily correlate with the degree of air pollution. Our recent measurements of PM_2.5_ from Punjab, Haryana and Delhi national capital region clearly show air pollution is in severe category in most days of late October and mid-November in the southern Punjab region and its neighbourhoods^10,36^.

### Implications

The findings of the present study highlight a critical need for targeted public education and policy interventions in Punjab. Many residents underestimated the severity of air pollution and were unaware of the link between stubble burning and air quality or the associated health risks. The results also indicate that knowledge of health risks from air pollution and having family members with health problems influenced attitude toward stubble burning. Such attitude plays a role in the awareness and mindset shifts that take place in the early stages of behavior change^37^. These findings are expected to inform future educational campaigns which are essential to communicate the health impacts of air pollution and the role of stubble burning in exacerbating these issues. For example, campaigns could focus on illustrating the connection between air pollution and personal health through familiar examples, instead of delivering knowledge solely as abstract information. Community-based seminars and awareness programs could improve environmental health literacy and help households make more informed decisions. Providing better access to observation-based air quality information in real-time through simple and comprehensible channels could further enhance public understanding of the issue^26^. Additionally, policies aimed at addressing environmental issues rely heavily on public awareness and support^32^. Raising awareness about the benefits of adopting sustainable agricultural practices and implementing stricter enforcement mechanisms could reduce burning and improve air quality and public health outcomes in the long run. Because there are smaller domestic burning sources which should also be eliminated in order to achieve more livable air quality in the urban areas, and that can be achieved only by participation of all residents. This is a lesson emerged from COVID-19 period when the shutting down all industrial sources did not meet the WHO’s safe air quality standards for Delhi^38^.

### Limitations

The present study has some limitations that should be acknowledged. First, random sampling was not feasible due to the COVID-19 pandemic and intermittent lockdowns during the period our survey. As a result, subjects were recruited based on geographic location, accessibility, and villagers’ willingness to participate. Second, while the survey was intended to be cross-sectional, its duration extended over six months due to unexpected delays caused by new waves of COVID-19. These delays may have influenced perceptions of air pollution and its health risks due to variations in air quality and pandemic-related circumstances during the survey period. Third, some respondents did not answer all the items in the interview schedule, leading to the exclusion of certain responses. This could have affected the quality and comprehensiveness of the analysis. Despite these limitations, the present study provides valuable insights into public perceptions of air pollution and health risks associated with stubble burning in Punjab.

## Conclusions

The present study offers significant insights into the perceptions of air pollution from stubble burning and its associated health risks among households in Punjab. Our findings reveal a disconnect between the recognized severity of air pollution in Delhi and the understanding of its causes, particularly the role of stubble burning in neighboring states. Although households with health issues were more likely to recognize the harmful effects of smoke from stubble burning, a substantial portion of the population remained unaware of the health risks associated with it. Additionally, the lack of significant associations between district-level fire counts or burnt areas and perception variables highlights the need for more robust and localized air quality data to better understand public perceptions. Addressing stubble burning and the resulting air pollution in Punjab requires a comprehensive approach involving various stakeholders. Enhancing public understanding of the link between better air quality and public health is essential. Educational initiatives, policy interventions, and stricter enforcement of sustainable agricultural practices could contribute to improved air quality and long-term public health benefits in the region.

## Methods

This study constitutes a part of an interview survey conducted in rural areas of Punjab starting in 2020 to examine stubble burning and human health impact within the framework of a project entitled “An Interdisciplinary Study toward Clean Air, Public Health and Sustainable Agriculture: The Case of Crop Residue Burning in North India”^39^. The survey protocol was approved by the Institutional Ethics Committee of RIHN (No. 2020-6) and the Kyoto University Engineering Research Ethics Committee (No. 202008) in February 2020, before the spread of COVID-19 in India. However, considering the subsequent COVID-19 situation, we carefully discussed its feasibility and timing of the survey with RIHN and the Center for International Projects Trust (CIPT), the NPO responsible for the survey, during the COVID-19 pandemic. The survey was conducted once the lockdown in India was lifted. Preventive measures such as physical distancing, wearing face masks, and hand sanitization were implemented to minimize infection risk during the survey. The research methods were carried out in accordance with the Declaration of Helsinki.

In the survey, CIPT enumerators provided an oral explanation along with the information sheet, and the survey was conducted only if the participants provided their informed consent. Additionally, participants were informed that they were not required to answer any questions they did not wish to respond to. Given the low literacy rate in the target area, informed consent was obtained verbally, but signatures were not required.

### Sampling and data collection

The survey was conducted in 22 districts of Punjab between August 2020 and January 2021. Multistage stratified random sampling was applied to select the households. At the first stage, two development blocks were chosen from each district through simple random sampling without replacement to achieve state-wide geographical coverage. At the second stage, one village was selected from each block that was medium-sized in terms of population, cultivated land area, and availability of basic agricultural infrastructure. A list of all farming households and their landholding sizes was prepared using village records and key informants. Stratified sampling was then used to select 50 households across different landholding categories—marginal, small, medium, and large. Prior to actual data collection, enumerators from CIPT assessed the supportiveness of key village leaders and the willingness of households to cooperate with the survey. When 50 farmers could not be selected from a single village, additional neighboring villages were included. Enumerators fluent in the local language interviewed householders after obtaining informed consent. All enumerators were trained beforehand to ensure respondents understood the questions and answered them rationally.

### Assessment of perceptions of air pollution and its health risks

We prepared a semi-structured interview schedule to assess perceptions of air pollution, health risks, and stubble burning. It consisted of several questions to gain a better understanding of the multiple dimensions of health risk perception: “What do you think about air pollution in Delhi/Punjab?” (perceived air pollution in Delhi/Punjab) on a 4-point Likert scale from “Not a problem,” “A minor problem,” “A moderate problem,” and “A severe problem”. As residents’ perceptions of air quality in Punjab can vary by individuals, we also asked those in Delhi which is often highlighted in the media for its serious air pollution. Comparing the perceptions of air quality in Punjab and Delhi can provide insight into baseline awareness. Other questions include: “Do you think smoke from stubble burning affects you and your family’s health?” (perceived health risks of air pollution) on a 3-point Likert scale from “No,” “Yes, a little,” and “Yes, very much”; “What do you think about stubble burning in Punjab?” (attitude toward stubble burning) on a 4-point Likert scale from “Not interested” to “Big problem, and have to stop now.” The interview schedule also included questions asking about the causes of air pollution in Delhi.

We also collected the following information from respondents: age, sex, education level, smoking habit, number of household members, annual income, health conditions of the respondents or household members during the stubble burning season/other season; and knowledge about health risks of air pollution using the question “Did you know air pollution increases the risk of respiratory and cardiovascular diseases?” (on a 3-point Likert scale from “No,” “Yes, a little,” and “Yes, very much.”

### District-specific data

Previous studies have reported that air pollution levels in living areas affect residents’ perceptions of air quality and health risks^18^. However, ground-level air pollution monitoring data in Punjab is scarce. As a proxy for air pollution levels due to stubble burning, monthly fire detection counts for each district during October and November of 2018 and 2019 were obtained from Visible Infrared Imaging Radiometer Suite (VIIRS) active fire data provided by NASA’s Fire Information for Resource Management System. Districts were categorized into two levels based on the 75th percentile of fire detection counts. District-specific estimates of burnt areas were derived from information obtained by village representative respondents. The information included the cultivated areas of rice in the kharif (rainy) season and wheat in the rabi (dry) season, as well as the extent to which farmers cultivated without burning residue or practiced partial burning after the rice harvest.

### Data analysis

We initially summarized respondents’ characteristics and their responses regarding perceptions of air quality and health. To explore factors influencing each dimension of health risk perception (perceived air quality in Delhi/Punjab, perceived health risk, and attitude), multilevel logistic regression model with random-intercept was used^40^. The four dependent variables were dichotomized as follows:

- Perceived air pollution in Delhi/Punjabi: “Severe” vs. reference category (“No problem”, “Little”, or “Medium”).
- Perceived health risk of smoke from stubble burning: “Yes, very much” or “Yes, a little” vs. reference category (“No problem”).
- Attitude toward stubble burning: “Big problem, and have to stop now” vs. reference category (“No problem, and do not have to stop”, “Some problems, but cannot stop”, or “No interest”).

District was included as a random effect. Since there were very few female respondents and smokers, we restricted the analysis to non-smoking male respondents. Individual- and household-level explanatory variables (age and education level of respondents, health conditions of household members, and annual income of household) were included in the model as categorical variables. Age was divided into three groups (< 40 years, 40–59 years, ≥ 60 years). Education level was categorized as “Primary or lower,” “Secondary and diploma,” and “Graduate and above.” Annual income was an ordered categorical variable divided into four levels based on the 25th, 50th, and 75th percentiles of annual household incomes. Health conditions of household members were categorized as “well” if no one was sick during both stubble burning and non-stubble burning seasons; otherwise, it was categorized as “sick.” Knowledge about health risks of air pollution was dichotomized as “No” if the respondent’s answer was “No problem”; otherwise, it was categorized as “Yes.” We also explored the effects of district-level characteristics, namely the level of fire detection counts and proportion of burnt areas. Odds ratios (ORs) with 95% confidence intervals (CIs) were calculated to estimate the strength of association between each explanatory variable and the outcome variable, adjusting for covariates included in the model. An OR > 1 suggests increased odds of the outcome compared to the reference category, whereas an OR < 1 suggests decreased odds.

We conducted a sensitivity analysis using a fixed-effects model that included district as dummy variables. In this model, we excluded hot spot numbers and proportion of burnt areas as they are district-level variables.

All analyses were performed using R (version 3.5.1).

## Supplementary Information

Below is the link to the electronic supplementary material.

Supplementary Material 1

## Data Availability

All data will be made available, following the acceptance of this article, from the Research Institute for Humanity and Nature on reasonable request. Researchers can refer to the corresponding author.
