# Supplementary material for: Perceptions of air pollution from stubble burning and its health risks in Punjab, India
Source: Sci Rep. 2025 Oct 27;15:37455. doi: 10.1038/s41598-025-21235-8 (PMC12559406; doi:10.1038/s41598-025-21235-8)
Supplement: Supplementary file 1 — Supplementary Material 1 [file 41598_2025_21235_MOESM1_ESM.pdf]

# Perceptions of air pollution from stubble burning and its health risks—a cross-sectional survey in Punjab, India

Zhesi YANG[1,2], Kayo UEDA[3,1]*, Tomohiro UMEMURA[4], Kazunari ONISHI[5], Hiroaki TERASAKI[6], Tomoki NAKAYAMA[7], Yutaka MATSUMI[1,8], Kamal VATTA[9], Hikaru ARAKI[1], Sachiko HAYASHIDA[1], Prabir K. PATRA[1, 10]

[1]Research Institute for Humanity and Nature

[2] Department of Global Health Policy, Graduate School of Medicine, The University of Tokyo

[3] Department of Hygiene, Graduate School of Medicine, Hokkaido University

[4] Aichi Medical University School of Medicine

[5] Division of Environmental health, Graduate School of Public Health, St Lukes International University

[6] Faculty of Engineering, University of Fukui

[7] Faculty of Environmental Science, Nagasaki University

[8] Institute for Space-Earth Environmental Research, Nagoya University, Nagoya

[9] Department of Economics and Sociology, Punjab Agricultural University

[10] Research Institute for Global Change, JAMSTEC

## Supplementary information

| | |
|---|---|
| Figure S1 | The proportion of each response for the question “How do you think about air pollution in Delhi?” and “How do you think about air pollution in Punjab?” |
| Figure S2 | Proportion of the burned area for each district in 2020 and the count of hotspots for each district during October and November in 2019 in each district |
| Table S1 | Sensitivity analysis: Odds ratios of individual factors on perception of air quality and its health risks with district fixed effects |

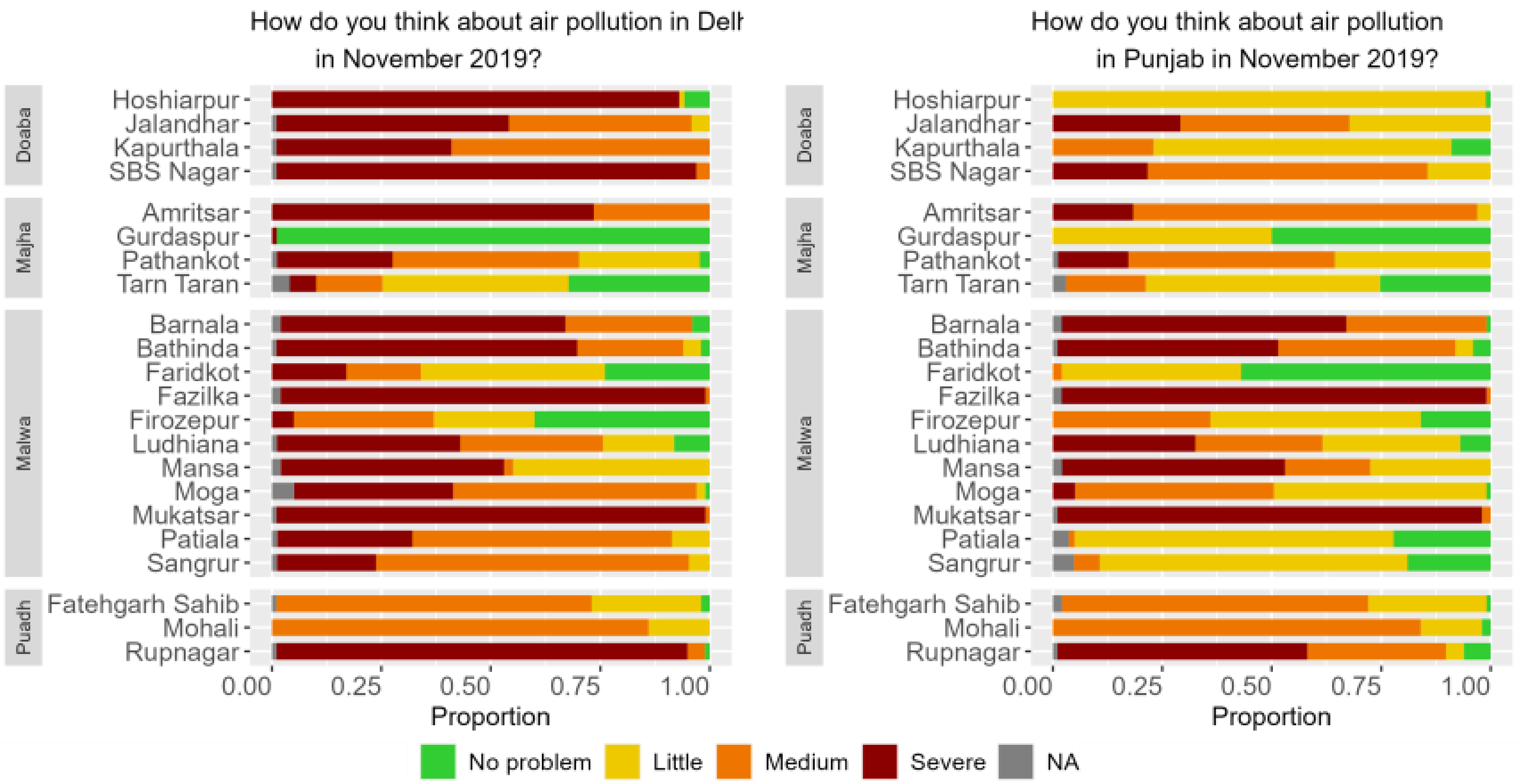


Figure S1. The proportion of each response for the question "How do you think about air pollution in Delhi?" (left) and "How do you think about air pollution in Punjab?" (right)

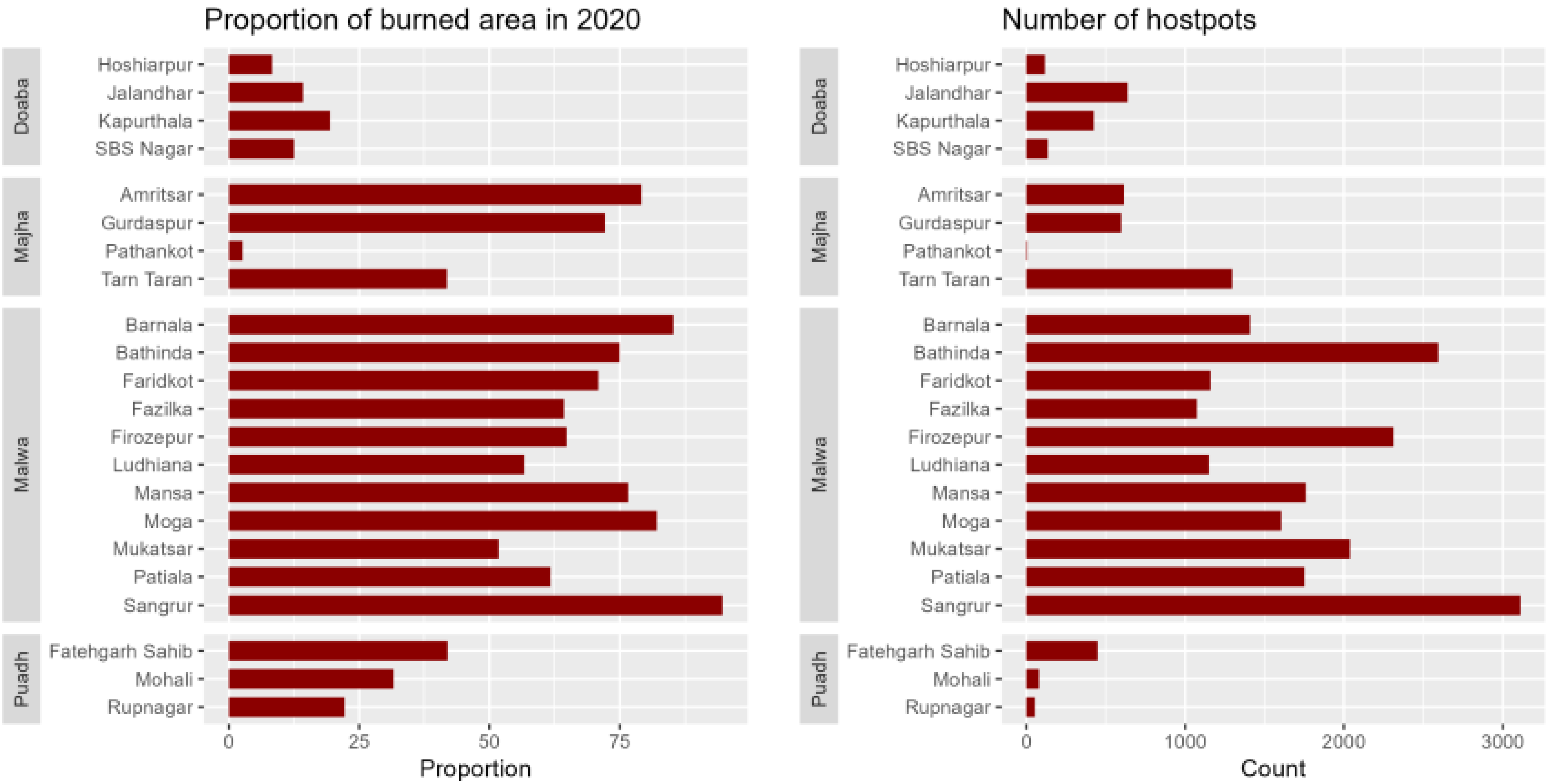


Figure S2. The proportion of the burnt area for each district in 2020 (left) and the fire detection counts for each district during October and November in 2019 (right) for each district

Table S1. Sensitivity analysis: Odds ratios of individual factors on perception of air quality and its health risks with district fixed effects

| Explanatory variable | Perceived air pollution in Delhi | Perceived air pollution in Punjab | Perceived health risk of smoke from stubble burning | Attitude toward stubble burning |
|---|---|---|---|---|
| | "Severe" vs "No problem" / "Little" / "Medium" | | "Yes, very much" / "Yes a little" vs "No problem" | "Big problem, and have to stop now" vs other responses |
| Individual-/household-level variables | | | | |
| Age | | | | |
| Less than 40 year (Reference) | 1 | 1 | 1 | 1 |
| 40-59 year | 1.49 (1.03, 2.15)* | 1.76 (1.10, 2.82)* | 1.46 (0.90, 2.38) | 0.72 (0.51, 1.02) |
| 60 year and over | 1.41 (0.93, 2.15) | 1.74 (1.03, 2.92)* | 0.87 (0.49, 1.54) | 0.78 (0.52, 1.18) |
| Educational status | | | | |
| Primary (Reference) | 1 | 1 | 1 | 1 |
| Secondary/diploma | 1.23 (0.91, 1.65) | 0.76 (0.54, 1.08) | 0.91 (0.60, 1.39) | 1.21 (0.90, 1.62) |
| Graduate/above | 1.30 (0.65, 2.56) | 1.78 (0.79, 4.00) | 0.89 (0.37, 2.14) | 1.25 (0.67, 2.32) |
| Household members | | | | |
| 1 or 2 (Reference) | 1 | 1 | 1 | 1 |
| 3 or 4 | 0.72 (0.38, 1.39) | 1.51 (0.69, 3.29) | 0.89 (0.36, 2.17) | 0.74 (0.38, 1.43) |
| 5 or 6 | 0.96 (0.50, 1.85) | 1.29 (0.59, 2.85) | 0.61 (0.24, 1.52) | 0.58 (0.30, 1.12) |
| 7 and over | 0.78 (0.38, 1.58) | 0.80 (0.34, 1.90) | 0.63 (0.23, 1.69) | 0.48 (0.24, 0.98)* |
| Household income | | | | |
| -144940 (Reference) | 1 | 1 | 1 | 1 |
| 144941-300000 | 0.78 (0.54, 1.14) | 0.80 (0.53, 1.23) | 1.32 (0.80, 2.20) | 0.85 (0.59, 1.23) |
| 300001-600000 | 0.69 (0.46, 1.03) | 1.09 (0.68, 1.73) | 1.15 (0.66, 2.00) | 0.87 (0.59, 1.30) |
| 600001- | 0.89 (0.58, 1.36) | 0.89 (0.53, 1.5.0) | 0.83 (0.45, 1.53) | 1.05 (0.70, 1.58) |
| Health condition of household | | | | |
| Well (Reference) | 1 | 1 | 1 | 1 |
| Sick | 0.82 (0.45, 1.48) | 1.27 (0.62, 2.61) | 38.89 (13.64, 110.92)* | 2.13 (1.14, 4.01)* |
| Knowledge | | | | |
| No (Reference) | 1 | 1 | 1 | 1 |
| Yes | 2.00 (1.46, 2.75)* | 1.28 (0.84, 1.94) | 24.00 (15.06, 38.24)* | 3.26 (2.33, 4.55)* |

* $P<0.05$
